# Dependency and health utilities in stroke: Data to inform cost-effectiveness analyses

**DOI:** 10.1177/2396987316683780

**Published:** 2017-03-01

**Authors:** Myzoon Ali, Rachael MacIsaac, Terence J Quinn, Philip M Bath, David L Veenstra, Yaping Xu, Marian C Brady, Anita Patel, Kennedy R Lees

**Affiliations:** 1 Institutes of Cardiovascular and Medical Sciences, Queen Elizabeth University Hospital, Glasgow, UK; 2 Institutes of Cardiovascular and Medical Sciences, Glasgow Royal Infirmary, Glasgow, UK; 3 Stroke Trials Unit, Division of Clinical Neuroscience, University of Nottingham, Nottingham, UK; 4 University of Washington, Seattle, WA, USA; 5 Genentech Inc., South San Francisco, CA, USA; 6 NMAHP Research Unit, Glasgow Caledonian University, Glasgow, UK; 7 Centre for Primary Care & Public Health, Blizard Institute, Queen Mary University of London, London, UK; 8 Institute of Cardiovascular & Medical Sciences, University of Glasgow, BHF Cardiovascular Research Centre, Glasgow, UK

**Keywords:** Stroke, trial, health utility, EQ-5D, cost effectiveness, modified Rankin Scale, quality of life

## Abstract

**Introduction:**

Health utilities (HU) assign preference weights to specific health states and are required for cost-effectiveness analyses. Existing HU for stroke inadequately reflect the spectrum of post-stroke disability. Using international stroke trial data, we calculated HU stratified by disability to improve precision in future cost-effectiveness analyses.

**Materials and methods:**

We used European Quality of Life Score (EQ-5D-3L) data from the Virtual International Stroke Trials Archive (VISTA) to calculate HU, stratified by modified Rankin Scale scores (mRS) at 3 months. We applied published value sets to generate HU, and validated these using ordinary least squares regression, adjusting for age and baseline National Institutes of Health Stroke Scale (NIHSS) scores.

**Results:**

We included 3858 patients with acute ischemic stroke in our analysis (mean age: 67.5 ± 12.5, baseline NIHSS: 12 ± 5). We derived HU using value sets from 13 countries and observed significant international variation in HU distributions (Wilcoxon signed-rank test *p* < 0.0001, compared with UK values). For mRS = 0, mean HU ranged from 0.88 to 0.95; for mRS = 5, mean HU ranged from −0.48 to 0.22. OLS regression generated comparable HU (for mRS = 0, HU ranged from 0.9 to 0.95; for mRS = 5, HU ranged from −0.33 to 0.15). Patients’ mRS scores at 3 months accounted for 65–71% of variation in the generated HU.

**Conclusion:**

We have generated HU stratified by dependency level, using a common trial endpoint, and describing expected variability when applying diverse value sets to an international population. These will improve future cost-effectiveness analyses. However, care should be taken to select appropriate value sets.

## Introduction

Changing population demographics will increase stroke prevalence and healthcare burden.^1^ With technological advances such as mechanical thrombectomy, and finite healthcare budgets, it is increasingly important to consider not just efficacy of new interventions but also the cost-effectiveness.^2^

Cost-effectiveness analyses are often based on the number of quality-adjusted life years (QALYs) that are gained from implementing a treatment. QALYs conveniently provide a combined estimate of both length and quality of life and can be used across a broad range of conditions, treatments and settings. Calculation of QALYs is dependent on (a) reliable measurements of patients’ health-related quality of life on at least two occasions and (b) the availability of accurate health utility (HU) estimates, which define and assign preference weights to each possible health state. HU are represented on a scale of <0 to 1, with 0 indicating equivalence with death, 1 representing perfect health, and negative values indicating states considered worse than death.

HU can be derived using diverse health state measures (e.g. the European Quality of Life Scale (EQ-5D-3L), the Health Utilities Index (HUI)^3^ and the Assessment of Quality of Life (AQoL)^4^); by various elicitation methods (Standard Gamble, Time Trade Off (TTO) and Visual Analogue Scale (VAS)^5^); and from various elicitation sources or populations. Value sets are usually collected from the general population. They exist for a range of different countries and describe preference weights for a particular health state.

For studies such as decision modelling that rely on existing sources of HU and include stroke as a possible health state, accurate HU must be generated.^6^ Currently for stroke, variation exists in the choice of elicitation method, and the generated HU show diversity within stroke as a condition.^2^ Stroke is characterised by a spectrum of functional outcomes; it is unfortunate that some calculated stroke HU have described only limited functional outcome states.^6^ Existing studies have described population characteristics for patients with HU < 0,^7^ have described methods to translate functional states into EQ-5D-3L utility values,^8^ have examined diversity in quality of life responses from participants from various countries, and have examined proxy respondents compared with self-reported outcomes.^9^ For international stroke trials, HU estimates derived from a single country may not be applicable to all available trial data. There are limited international data to describe the range of expected HU across all possible levels of function, generated using a range of value sets. We sought to better inform future cost-effectiveness analyses that require HU estimates for stroke, by generating international HU based on European Quality of Life Scale (EQ-5D-3L) scores at a common acute stroke trial endpoint (3 months following stroke), and mapped across a spectrum of functional outcomes, assessed using the modified Rankin Scale (mRS).

## Methods

### Data

We conducted retrospective analyses of pooled, anonymised, patient-level data from the Virtual International Stroke Trials Archive (VISTA)^10^ on demography, (age, sex, medical history), neurological impairment (National Institutes of Health Stroke Scale score (NIHSS)), functional outcome (mRS), Quality of Life (EQ-5D-3L) and country of enrolment. The mRS is a 7-point observational scale that describes level of dependency, and ranges from 0 (no symptoms at all) to 6 (dead). The EQ-5D-3L is a standardised measurement tool for health-related quality of life and includes domains of mobility, self-care, usual activities, pain/discomfort and anxiety/depression. It can also be completed by proxy for people unable to complete the questionnaire themselves.

### HU generation

We utilised published country-specific preference weights (value sets)^11–23^ to calculate HU. Each published value set was elicited from general population samples from the respective countries, using the Time Trade off (TTO) method. These value sets were applied in turn to individual-level EQ-5D-3L health state descriptions based on the five domains of mobility, self-care, usual activities, pain/discomfort and anxiety/depression for patients in our dataset (online Supplement I).

We applied each published value set to our data, stratifying by mRS score at 3 months to illustrate expected variation when applying any single value set to an international trial population, as commonly occurs in cost effectiveness analysis. We examined potential differences in the distributions of HU according to the value set applied, with the Wilcoxon signed-rank test, and using HU generated from the UK value set as a reference population. Supplementary analyses applied each published value set to the country-specific population from which it was derived; if populations existed where no country-specific value set was available, we applied the value set of the nearest neighbouring country.

### Validation

Ordinary least squares (OLS) regression^24,25^ is recommended as a method of estimating unknown parameters (such as HU) from existing data (e.g. mRS).^26^ OLS Regression examines error: the differences between predicted outcomes and reality, and attempts to fit a line through the data that minimises the sum of the squared errors. This method was also previously described by Rivero-Arias et al.^8^ We used OLS regression to generate an equation to estimate HU based on mRS scores from our international population. We examined the proportion of variation in HU that was explained by mRS, adjusting for patients’ age and baseline NIHSS. The National Institute for Health and Clinical Excellence (NICE) caution against over-fitting covariates in an OLS regression; age and NIHSS were selected due to the strength of their association with post-stroke outcomes in our dataset (*p* < 0.0001).

For this regression analysis, we applied published value sets from the USA, UK, Spain, Germany, China and Poland. These value sets were selected as they were generated using the most robust sample sizes,^11^ were published in the EQ-5D-3L inventory and user guide, represented countries that were typically included in international multicentre RCTs, and/or represented areas where emerging stroke research datasets warranted the generation of robust HU estimates. Performance was assessed using goodness of fit (adjusted *R*-squared values). We described the clinical and demographic characteristics of our population to inform generalisability for application to other clinical stroke populations.

## Results

We identified and extracted eligible data on 4946 patients (mean age: 68.8 ± 12.6 years, 2231 (45%) female, baseline NIHSS: 12 ± 9; Table 1) for whom assessment of EQ-5D-3L and mRS had been performed. Our analysis dataset comprised patients who were alive and had complete mRS and EQ-5D-3L scores at 3 months following stroke; by 3 months 817 (17.0%) patients had died; complete data on EQ-5D-3L (76.4% subject and 21.8% proxy respondents) and mRS were available for 3858 patients (mean age: 67.5 ± 12.5, baseline NIHSS: 12 ± 5) and missing for 271. Thirty-six countries were represented in our analysis dataset (online Supplement II).

**Table 1.** Baseline demography (*n* = 4946 participants).

Variable	Value
Age (years); mean (SD)	68.8 (12.6)
Baseline NIHSS; median (IQR)	12 (9)
Gender (female); *n* (%)	2231 (45.1%)
RTPA (yes); *n* (%)	1915 (38.7%)
Diabetes (yes); *n* (%)	1135 (22.9%)
Hypertension (yes); *n* (%)	3665 (74.1%)
Atrial fibrillation (yes); *n* (%)	1271 (25.7%)
Previous stroke (yes); n (%)	979 (19.8%)
Transient ischaemic attack (yes); *n* (%)	409 (8.3%)
Myocardial infarction (yes); *n* (%)	641 (13.0%)
Congestive heart failure (yes); *n* (%)	467 (9.4%)

Age and initial stroke severity by NIHSS were largely comparable across countries having a sample size of more than 50 patients. Medical history and use of thrombolytics varied by country of enrolment particularly in those countries that enrolled fewer patients (Argentina, Brazil, Chile, Finland, Greece, Hong Kong, Italy, Malaysia, Mexico, Netherlands, Norway, South Africa, South Korea and Sweden; online Supplement II).

### HU estimates

After applying each published value set to our international dataset in turn, for mRS = 0, mean HU ranged from 0.88 to 0.95; for mRS = 5, mean HU ranged from −0.48 to 0.22 (Table 2). HU for mRS = 5 were perceived as corresponding to a health state that was worse than death when applying value sets from Singapore (−0.48), Spain (−0.34), and the UK (−0.15). Similar HU ranges were observed when excluding proxy responses on EQ-5D-3L (online Supplement III). The Wilcoxon signed-rank test revealed significant differences between the HU distributions generated using each country’s value set, when compared with those generated using the UK value set (*p* < 0.0001 for each country; Table 2).

**Table 2.** Mean HU derived using EQ-5D-3L, stratified by mRS.

Value set applied to entire international dataset	Modified Rankin Scale score at 3 months						
	0 (*n* = 529)	1 (*n* = 866)	2 (*n* = 633)	3 (*n* = 669)	4 (*n* = 825)	5 (*n* = 336)	Wilcoxon signed-rank test for differences in distributions of HU, relative to UK distribution (*p* values)
Australia	0.93 (0.13)	0.86 (0.16)	0.76 (0.17)	0.61 (0.21)	0.35 (0.27)	0.02 (0.18)	<0.0001
China	0.92 (0.12)	0.84 (0.15)	0.73 (0.16)	0.58 (0.17)	0.37 (0.20)	0.15 (0.16)	<0.0001
Denmark	0.91 (0.15)	0.83 (0.16)	0.73 (0.16)	0.61 (0.19)	0.37 (0.19)	−0.02 (0.27)	<0.0001
Germany	0.95 (0.12)	0.90 (0.14)	0.83 (0.18)	0.68 (0.23)	0.38 (0.27)	0.09 (0.18)	<0.0001
Netherlands	0.91 (0.16)	0.83 (0.18)	0.73 (0.19)	0.59 (0.23)	0.35 (0.25)	0.12 (0.21)	<0.0001
Poland	0.94 (0.11)	0.89 (0.12)	0.81 (0.14)	0.70 (0.20)	0.43 (0.29)	0.06 (0.27)	<0.0001
Singapore	0.88 (0.21)	0.74 (0.28)	0.51 (0.30)	0.23 (0.32)	−0.16 (0.33)	−0.48 (0.22)	<0.0001
South Korea	0.94 (0.10)	0.88 (0.11)	0.80 (0.12)	0.69 (0.15)	0.42 (0.25)	0.09 (0.18)	<0.0001
Spain	0.93 (0.14)	0.85 (0.18)	0.72 (0.21)	0.51 (0.28)	0.09 (0.36)	−0.34 (0.23)	<0.0001
UK	0.90 (0.17)	0.82 (0.19)	0.70 (0.21)	0.53 (0.26)	0.20 (0.31)	−0.15 (0.23)	–
USA	0.92 (0.12)	0.85 (0.14)	0.77 (0.14)	0.64 (0.17)	0.41 (0.22)	0.14 (0.15)	<0.0001
Zimbabwe	0.92 (0.12)	0.85 (0.13)	0.75 (0.13)	0.63 (0.15)	0.45 (0.19)	0.22 (0.17)	<0.0001

Note: HU displayed as mean (SD) and generated using available, published value sets.

Online Supplement IV describes HU generated by applying country-specific value sets to appropriate sub-populations. We observed that for mRS = 0, the mean HU estimates ranged between 0.81 and 0.98. For mRS = 5, mean HU ranged between −0.48 and 0.27.

### Validation

After applying OLS regression, mRS scores at 3 months accounted for 65–71% of the variation in the generated HU estimates. The HU generated using OLS regression were consistent with those generated by applying each value set to the analysis dataset (Table 3). For mRS = 0 mean HU ranged between 0.9 and 0.95; for mRS = 1, mean HU ranged between 0.81 and 0.9, and for mRS = 5, mean HU ranged between −0.33 and 0.15.

**Table 3.** Mean HU calculated using OLS regression, stratified by mRS.

Value set applied to analysis dataset		Mean HU, by modified Rankin Scale score at 3 months					
	Adjusted *R*^2^	0	1	2	3	4	5
China	68.8	0.92	0.84	0.73	0.58	0.37	0.15
Germany	65.8	0.95	0.90	0.83	0.69	0.38	0.09
Poland	63.7	0.94	0.89	0.81	0.70	0.44	0.07
Spain	71.2	0.92	0.84	0.72	0.51	0.09	−0.33
UK	65.0	0.90	0.81	0.70	0.53	0.20	−0.14
USA	67.4	0.92	0.84	0.72	0.51	0.09	−0.33

## Discussion

We generated exemplar international acute stroke HU based on published value sets, describing case mix and stratifying by mRS at 3 months to better inform future cost-effectiveness analyses. The range of observed HU generated by applying each published value set to our international population was similar to those generated when using OLS regression, and when excluding proxy responses.

For mRS of 0, the mean HU ranged between 0.88 and 0.95, indicating that even though these patients were by definition asymptomatic, there were extraneous influences on the individual that affected perception of their health state. mRS states can be assigned on the basis of physical disability, cognitive impairment or a combination of both. Furthermore, the EQ-5D-3L has five domains and within any mRS level, patients can exhibit variation in which EQ-5D-3L domains have been affected. Therefore, it is possible for considerable variation to exist in HU estimates within a single mRS level. It is also possible that scoring errors or inconsistencies on mRS and on EQ-5D-3L contribute to this variation.

We observed that application of different value sets resulted in significantly different distributions of HU (compared with UK values). Since value sets vary according to country, heterogeneity in HU is expected when these diverse value sets are applied to a single multi-centred international trial population. The variation in value sets could arise from differing access to, and levels of health care services available, as well as differing cultural perceptions of disability across participating countries. This issue applies to both health state measurement and health state valuation, and should be taken into consideration when selecting appropriate value sets to inform cost-effectiveness of an intervention.

Guidance is needed on the application of appropriate value sets for pooled analyses of international populations. The application of one value set to an international population is commonly practiced, often as a matter of convenience or because this approach is applied to the corresponding cost data. However, this approach has some limitations; between country differences exist in health-related QoL, costs of healthcare, the degree of social support available and cultural perceptions of disability. These differences are not captured when applying a single value set to an international population. Application of country-specific value sets increases the relevance of the generated HU to each country, but creates problems for pooling of data for analyses (which is often necessary to preserve sample size). Our supplementary analysis still necessitated the application of a single value set to multiple neighbouring countries (online Supplement IV). For example data from Germany, Switzerland, Italy, and Greece were analysed using the German value set. The latter countries have strong family support for stroke survivors, and the application of German preference weights to these participants may not fully capture subtle differences in health perceptions within the same mRS level. Similar issues arise with the application of the USA value set to Central and South American countries. Our application of country-specific value sets to appropriate populations (online Supplement IV) highlights a challenge when dealing with smaller subgroups. We observed that when applying a Nordic value set to Nordic countries, HU were greater for mRS = 2 (0.92), than for mRS = 0 (0.9). Similarly, applying the UK value set to UK participants, HU for mRS = 1 (0.9) was greater than HU for mRS = 0 (0.81). This difference could be attributed to participant heterogeneity. Pooling data hides the country-level issues, and results are often not specifically relevant to any participating counties, while sub-group analyses carry analytical deficiencies. There is often a trade-off between the availability and appropriateness of value sets for use in an international population, and preservation of a large enough sample size on which inferences can be made on health perception and cost-effectiveness.

Debate also exists over the appropriate participant population from which to derive HU estimates.^6^ Those at risk of stroke are traditionally seen to be more suited to inform decisions from a patient’s perspective.^27^ However preference values derived from hypothetical scenarios may not be valid predictors of the preferences associated with actual experienced health states^28^; stroke survivors typically assign higher values to health states than those at risk of stroke, or healthy participants.^6^ Nevertheless, preference weight estimates from the general population are recommended when assessing cost-effectiveness from a societal perspective.^6,27^

We described HU generated from application of both single and country-specific value sets to an international population. Previous studies have utilised a single country’s value set,^7^ described HU generated from a range of stroke and non-stroke populations, or stratified by broad categories of disability (minor stroke = mRS 2–3, major stroke = mRS 4–5).^6^ Previous estimates elicited from stroke survivors using the EQ-5D-3L described utilities of 0.71 and 0.32 for minor and major stroke, respectively.^6^ This contrasts with our findings where we observed a much wider HU range for the transition from mRS 2 to 5 (from 0.83 to −0.48). Although our data give HU values that differ from previously published estimates, our results are still within a range that would seem credible based on previous work.^6^ Furthermore, our generation of HU based on mapping approaches (Table 3) are consistent overall with HU generated from a prior study by Rivero-Arias et al.,^8^ though it should be noted that their study generated HU at different time points post-stroke.

Our approach to HU had a number of strengths. Our data are representative of the range of respondents that are typical in acute stroke RCTs. We employed OLS regression^24^ to validate our estimates. We used a generic patient reported outcome measure (EQ-5D-3L) that has been specified as a preferred method of utility measurement in clinical trials.^24^ Our analysis includes a much larger and more geographically diverse patient population than examined in previous studies. Baseline data suggest that included patients are broadly representative of acute stroke trial cohorts.

A limitation of our study is that perspectives on health states may change according to the time since stroke, and the values elicited based on EQ-5D-3L may not fully capture information from some patient subgroups such as those with communication problems. Those with cognitive or visual problems may rely on proxies to complete the EQ-5D-3L and thus their views may not be accurately represented. However, in our analysis dataset, 76.4% of EQ-5D-3L responses were elicited from stroke survivors themselves. Additionally, we analysed data only from those who had complete scores on all domains of EQ-5D-3L at 3 months; this may have biased the sample sizes available at higher levels of dependence. Furthermore, our data are based on an acute stroke clinical trial population. The HU generated for each stratum of mRS are therefore based on the experiences of a subgroup of the general stroke population. Future work could examine the generalisability of the HU generated in our population to general stroke population, and additional work is needed to examine the minimum sample size required for reliable country-specific HU generation.

Our study is based on acute stroke clinical trial data including information on dependency at a common endpoint, and involving patients from countries typically represented in acute stroke trials. Our findings can inform cost-effectiveness analyses of interventions in the acute stroke setting by providing conservative estimates of HU across a range of dependency levels; this may be of particular use to study designs reliant on secondary data sources, for example decision models. HU could feasibly be calculated in future studies through the collection of EQ-5D-3L data in parallel with common trial outcomes such as mRS.

As more people survive stroke with long term disability,^1^ cost-effectiveness analyses should take into consideration whether an intervention has longer-term benefits for stroke survivors. Generation of HU for various levels of dependency at longer time points post-stroke is desirable. Future research could also involve calculation of the adjustment factors needed to convert known mRS distributions to HU according to age and sex, to refine our current estimates.

## Supplementary Material

Supplementary material
