# Supplementary material for: Dependency and health utilities in stroke: Data to inform cost-effectiveness analyses
Source: Eur Stroke J. 2017 Mar 1;2(1):70–6. doi: 10.1177/2396987316683780 (PMC6027777; doi:10.1177/2396987316683780)
Supplement: Supplementary material [file ESO683780_supplemental_material.pdf]

Supplemental Material

Supplement I) Published TTO-based valuation models with country, reference and sample size from which they were elicited.

	TTO Value Set												
**Variable**	**UK[2]**	**Spain[5]**	**Netherlands[6]**	**Germany[7]**	**Zimbabwe[8]**	**USA[3]**	**Denmark[1]**	**Japan[9]**	**Poland[10]**	**South Korea[11]**	**China[12]**	**Singapore[13]**	**Australia[14]**
**N2 (Constant)**	-0.081	-0.024	-0.071	-0.001	-0.100	-	-0.114	-0.152	-0.049	0.050	-0.039	-	-
**N3**	-0.269	-0.291	-0.234	-0.323	-	-	-	-	-	0.050	-0.022	-0.2905	-
**MO2**	-0.069	-0.106	-0.036	-0.099	-0.056	-0.146	-0.053	-0.075	-0.052	0.096	-0.099	-0.1678	-0.080
**MO3**	-0.314	-0.430	-0.161	-0.327	-0.204	-0.558	-0.411	-0.418	-0.331	0.418	-0.246	-0.3040	-0.372
**SC2**	-0.104	-0.134	-0.082	-0.087	-0.092	-0.175	-0.063	-0.054	-0.054	0.046	-0.105	-0.1615	-0.109
**SC3**	-0.214	-0.309	-0.152	-0.174	-0.231	-0.471	-0.192	-0.063	-0.235	0.136	-0.208	-0.3465	-0.291
**UA2**	-0.036	-0.071	-0.032	-	-0.043	-0.140	-0.048	-0.044	-0.046	0.051	-0.074	-0.2555	-0.072
**UA3**	-0.094	-0.195	-0.057	-	-0.135	-0.374	-0.144	-0.133	-0.212	0.208	-0.193	-0.3209	-0.165
**PD2**	-0.123	-0.089	-0.086	-0.112	-0.067	-0.173	-0.062	-0.080	-0.057	0.037	-0.092	-0.1462	-0.085
**PD3**	-0.386	-0.261	-0.329	-0.315	-0.302	-0.537	-0.396	-0.194	-0.489	0.151	-0.236	-0.2291	-0.473
**AD2**	-0.071	-0.062	-0.124	-	-0.046	-0.156	-0.068	-0.063	-0.026	0.043	-0.086	-0.1501	-0.118
**AD3**	-0.236	-0.144	-0.325	-0.065	-0.173	-0.450	-0.367	-0.112	-0.207	0.158	-0.205	-0.2905	-0.424
**D1**	-	-	-	-	-	+0.140	-	-	-	-	-	-	-
**I2-sq**	-	-	-	-	-	-0.011	-	-	-	-	-	-	-
**I3**	-	-	-	-	-	+0.122	-	-	-	-	-	-	-
**I3-sq**	-	-	-	-	-	+0.015	-	-	-	-	-	-	-
**MO3_SC3**	-	-	-	-	-	-	-	-	-	-	-	-	0.061
**MO3_UA3**	-	-	-	-	-	-	-	-	-	-	-	-	-0.031
**MO3_PD3**	-	-	-	-	-	-	-	-	-	-	-	-	0.094
**MO3_AD3**	-	-	-	-	-	-	-	-	-	-	-	-	0.016
**SC3_UA3**	-	-	-	-	-	-	-	-	-	-	-	-	-0.050
**SC3_PD3**	-	-	-	-	-	-	-	-	-	-	-	-	0.100
**SC3_AD3**	-	-	-	-	-	-	-	-	-	-	-	-	0.104
**UA3_PD3**	-	-	-	-	-	-	-	-	-	-	-	-	0.032
**UA3_PD3**	-	-	-	-	-	-	-	-	-	-	-	-	0.060
**PD3_AD3**	-	-	-	-	-	-	-	-	-	-	-	-	0.186

N2-at least one 2 or 3, N3-At least one 3, MO2-Mobility=2, MO3-Mobility=3, SC2-Self Care=2, SC3-Self Care=3, UA2-UsualActivities=2, UA3-Usual Activities=3, PD2-Pain and discomfort=2, PD3-Pain and Discomfort=3, AD2-Anxiety and Depression=2 and AD3-Anxiety and Depression=3. For the USA, extra coefficients were added: D1-number of movements away from full health beyond the first, I2-The number of dimensions at level 2 beyond the first, I3-number of dimensions at level 3 beyond the first, I2-Sq-square of I2 and I3-Sq-square of I3. For the

Australian dataset, interactions added included MO3_SC3 (interaction between Mobility=3 and Self Care=3). For the Australian utility weights we used model 3b; model 3 with the constant included elicited unusual values so it was rejected in favour of the model without.

Supplement II) Case Mix at baseline, Stratified by Country of Inclusion

	Median [IQR]*				Freq (%)								
	Age	Baseline NIHSS[†]	mRS[‡] 90	EQ-5D-3L[§] Proxy	Male Sex	Left Hemisphere	Prior Stroke	Rt-PA[‖]	Hypertension	Diabetes	Prior MI[#]	AFIB[**]	CHF[‡‡]
**Australia/ New Zealand (n=82)**	71 [59,79]	13 [8,16]	3 [2,4]	17 (21)	39 (47.6)	40 (48.8)	8 (9.8)	38 (46.3)	60 (73.2)	11 (13.4)	13 (15.9)	24 (29.3)	9 (11)
**Austria (n=57)**	70 [57,77]	10 [7,15]	2 [1,4]	12 (21.1)	34 (59.7)	29 (50.9)	5 (8.8)	35 (61.4)	36 (63.2)	11 (19.3)	7 (12.3)	17 (29.8)	7 (12.3)
**Belgium (n=106)**	74 [65,79]	14 [9,19]	3 [2,4]	23 (21.9)	53 (50)	48 (45.3)	13 (12.3)	59 (55.7)	61 (57.6)	17 (16)	14 (13.2)	33 (31.1)	7 (6.6)
**Brazil (n=44)**	71 [56.5,79]	13 [8,20]	2 [0,4]	19 (44.2)	20 (45.5)	24 (54.6)	9 (20.5)	29 (65.9)	36 (81.8)	8 (18.2)	5 (11.4)	9 (20.5)	3 (6.8)
**Bulgaria (n=117)**	67 [60,72]	8 [7,11]	2 [1,3]	22 (18.8)	73 (62.4)	52 (44.4)	22 (18.8)	0 (0)	99 (84.6)	24 (20.5)	8 (6.8)	21 (18)	24 (20.5)
**Canada (n=298)**	72.5 [62,79]	12 [9,17]	3 [1,4]	80 (26.9)	155 (52)	129 (43.4)	56 (18.8)	201 (67.5)	223 (74.8)	62 (20.8)	45 (15.1)	70 (23.5)	23 (7.7)
**China (n=62)**	68.5 [56,76]	11 [7,15]	3 [2,4]	19 (31.2)	41 (66.1)	28 (45.2)	15 (24.2)	7 (11.3)	40 (64.5)	12 (19.4)	3 (4.8)	13 (21)	1 (1.6)
**Czech Republic (n=425)**	70 [60,76]	8 [7,11]	2 [1,3]	34 (8)	244 (57.4)	196 (46.1)	80 (18.8)	98 (23.1)	330 (77.7)	107 (25.2)	55 (12.9)	80 (18.8)	6 (1.4)
**France (n=86)**	64 [51,75]	14 [9,19]	3 [1,4]	7 (8.1)	49 (57)	44 (51.2)	8 (9.3)	42 (48.8)	47 (54.7)	15 (17.4)	8 (9.3)	23 (26.7)	4 (4.7)
**Germany (n=138)**	66 [58,77]	10 [8,13]	2 [1,4]	11 (8.1)	89 (64.5)	45 (32.6)	16 (11.6)	85 (61.6)	109 (79)	24 (17.4)	13 (9.4)	26 (18.8)	11 (8)
**Hungary (n=167)**	67 [59,75]	9 [8,12]	1 [1,3]	10 (6)	96 (57.5)	93 (55.7)	26 (15.6)	56 (33.5)	132 (79)	25 (15)	14 (8.4)	32 (19.2)	8 (4.8)
**Israel (n=133)**	69 [58,77]	11 [8,16]	3 [2,4]	49 (37.7)	74 (55.6)	66 (49.6)	29 (21.8)	36 (27.1)	98 (73.7)	44 (33.1)	18 (13.5)	27 (20.3)	13 (9.8)
**Philippines (n=95)**	63 [52,70]	10 [7,15]	2 [1,4]	30 (31.6)	40 (42.1)	51 (53.7)	22 (23.2)	2 (2.1)	77 (81.1)	26 (27.4)	5 (5.3)	16 (16.8)	4 (4.2)
**Poland (n=92)**	70 [60.5,76]	11.5 [9,15]	2 [1,3]	26 (28.3)	50 (54.4)	36 (39.1)	14 (15.2)	12 (13)	65 (70.7)	23 (25)	10 (10.9)	28 (30.4)	9 (9.8)
**Portugal (n=111)**	70 [59,77]	15 [9,18]	3 [2,4]	42 (39.3)	48 (43.2)	59 (53.2)	31 (27.9)	20 (18)	76 (68.5)	16 (14.4)	4 (3.6)	25 (22.5)	9 (8.1)
**Russia (n=87)**	68 [58,77]	8 [7,12]	2 [1,3]	21 (24.1)	54 (62.1)	51 (58.6)	24 (27.6)	0 (0)	79 (90.8)	9 (10.3)	18 (20.7)	25 (28.7)	3 (3.5)
**Singapore (n=56)**	66 [54,75]	15 [9,19]	3 [1,4]	32 (57.1)	34 (60.7)	26 (46.4)	8 (14.3)	2 (3.6)	34 (60.7)	18 (32.1)	5 (8.9)	10 (17.9)	2 (3.6)
**Slovak Republic**	70 [59,76]	8 [6,10]	1 [1,4]	2 (0.8)	135 (54.2)	109 (43.8)	58 (23.3)	25 (10)	218 (87.6)	63 (25.3)	29 (11.7)	50 (20)	2 (0.8)

**(n=249)**													
**Spain (n=287)**	72 [63,78]	14 [10,18]	3 [1,4]	100 (37.2)	161 (56.1)	124 (43.9)	40 (13.9)	141 (49.1)	175 (61)	59 (20.6)	11 (3.8)	63 (22)	15 (5.2)
**Switzerland (n=105)**	70 [61,77]	12 [7,17]	2 [1,4]	27 (27.8)	70 (66.7)	43 (41)	16 (15.2)	84 (80)	72 (68.6)	20 (19.1)	15 (14.3)	33 (31.4)	6 (5.7)
**Taiwan (n=53)**	75 [65,78]	12 [9,16]	3 [1,4]	22 (41.5)	34 (64.2)	22 (41.5)	19 (35.9)	13 (24.5)	40 (75.5)	11 (20.8)	5 (9.4)	11 (20.8)	4 (7.6)
**UK (72)**	70 [59.5,76]	15 [10,19]	3 [2.5,4]	14 (19.7)	36 (50)	41 (56.9)	13 (18.1)	39 (54.2)	36 (50)	6 (8.3)	5 (6.9)	17 (23.6)	3 (4.2)
**USA (n=792)**	70 [58,78]	12 [8,17]	2 [1,4]	163 (21)	420 (53)	376 (47.5)	200 (25.3)	447 (56.4)	633 (79.9)	203 (25.6)	143 (18)	211 (26.6)	121 (15.3)
**Argentina (n=16)**	65 [55.5,71]	11 [7,16]	3 [2,4]	4(25)	11 (68.8)	12 (75)	3 (18.8)	2 (12.5)	8 (50)	4 (25)	0 (0)	3 (18.8)	1 (6.3)
**Chile (n=10)**	72.5 [56,81]	15.5 [14,20]	2.5 [2,4]	3 (30)	5 (50)	7 (70)	2 (20)	6 (60)	6 (60)	0(0)	0 (0)	1 (10)	0(0)
**Finland (n=18)**	72 [59,76]	12.5 [9,16]	3 [1,4]	4 (22.2)	9 (50)	9 (50)	2 (11.1)	9 (50)	9 (50)	3 (16.7)	5 (27.8)	4 (22.2)	6 (33.3)
**Greece (n=24)**	72.5 [66.5,78]	10 [7,14]	3 [1,4]	6 (25)	11 (45.8)	9 (37.5)	5 (20.8)	1 (4.2)	20 (83.3)	7 (29.2)	1 (4.2)	9 (37.5)	1 (4.2)
**Hong Kong (n=25)**	71 [62,74]	12 [10,19]	4 [2,4]	10 (40)	15 (60)	9 (36)	3 (12)	1 (4)	18 (72)	7 (28)	0 (0)	10 (40)	0 (0)
**Italy (n=25)**	71 [61,78]	10 [7,12]	1 [0,3]	2 (10)	11 (44)	15 (60)	6 (24)	10 (40)	18 (72)	4 (16)	2 (8)	7 (28)	3 (12)
**Malaysia (n=12)**	62.5 [47,71]	11.5 [10,14.5]	3 [1.5,4.5]	4 (33.3)	8 (66.7)	6 (50)	1 (8.3)	0 (0)	7 (58.3)	5 (41.7)	0 (0)	0 (0)	0 (0)
**Mexico (n=24)**	66.5 [47.5,78.5]	11.5 [7,15]	2 [1,3]	9 (37.5)	9 (37.5)	12 (50)	7 (29.2)	8 (33.3)	11 (45.8)	6 (25)	1 (4.2)	2 (8.3)	0 (0)
**Netherlands (n=27)**	73 [65,75]	13 [9,16]	3 [2,4]	4 (14.8)	19 (70.4)	13 (48.2)	6 (22.2)	13 (48.2)	12 (44.4)	2 (7.4)	4 (14.8)	4 (14.8)	1 (3.7)
**Norway (n=4)**	71.5 [63,74.5]	17 [10,22]	3.5 [2,4.5]	3 (75)	3 (75)	1 (25)	0 (0)	3 (75)	3 (75)	0 (0)	0 (0)	1 (25)	0 (0)
**South Africa (n=43)**	57 [50,78]	10 [8,15]	1 [1,3]	7 (16.7)	25 (58.1)	19 (44.2)	5 (11.6)	4 (9.3)	28 (65.1)	8 (18.6)	2 (4.7)	13 (30.2)	1 (2.3)
**South Korea (n=32)**	36 [59,72]	11 [8,15.5]	2.5 [1,4]	8 (25.8)	10 (31.3)	13 (40.6)	6 (18.8)	15 (46.9)	19 (59.4)	10 (31.3)	3 (9.4)	10 (31.3)	2 (6.3)
**Sweden (n=19)**	79 [72,83]	14 [10,17]	3 [1,4]	8 (44.4)	9 (47.4)	9 (47.4)	2 (10.5)	4 (21.1)	5 (26.3)	4 (21.1)	7 (36.8)	7 (36.8)	1 (5.3)

*Interquartile Range

†National Institutes of Health Stroke Scale

‡Modified Rankin Scale

§European Quality of Life Scale

| | Recombinant Tissue Plasminogen Activator

[#] Myocardial Infarction

[**]Atrial Fibrillation

#Congestive Heart Failure

Supplement III) Mean HU derived using EQ-5D-3L (subject respondents only), applying each published value set to the international population, and stratified by mRS at 3 months (HU displayed as mean (StdDev))

Value Set applied across respondents-only population	Modified Rankin Scale Score at 3 months					
	**0 (n=516)**	**1 (n=840)**	**2 (n=571)**	**3 (n=508)**	**4 (n=488)**	**5 (n=82)**
Australia	0.93 (0.13)	0.86 (0.16)	0.76 (0.18)	0.61 (0.2)	0.39 (0.27)	0.02 (0.2)
China	0.92 (0.12)	0.85 (0.15)	0.73 (0.16)	0.59 (0.17)	0.40 (0.19)	0.15 (0.16)
Denmark	0.91 (0.15)	0.83 (0.16)	0.73 (0.16)	0.62 (0.18)	0.41 (0.29)	-0.00 (0.28)
Germany	0.95 (0.11)	0.90 (0.14)	0.83 (0.18)	0.69 (0.22)	0.42 (0.27)	0.09 (0.19)
Netherlands	0.91 (0.16)	0.83 (0.18)	0.73 (0.2)	0.60 (0.23)	0.39 (0.25)	0.12 (0.2)
Poland	0.94 (0.11)	0.89 (0.12)	0.81 (0.14)	0.70 (0.19)	0.47 (0.29)	0.07 (0.29)
Singapore	0.88 (0.21)	0.74 (0.28)	0.51 (0.3)	0.24 (0.31)	-0.10 (0.33)	-0.47 (0.2)
South Korea	0.94 (0.1)	0.88 (0.11)	0.80 (0.12)	0.69 (0.14)	0.46 (0.24)	0.10 (0.19)

Spain	0.93 (0.14)	0.85 (0.18)	0.72 (0.21)	0.52 (0.27)	0.15 (0.36)	-0.33 (0.24)
UK	0.90 (0.17)	0.82 (0.19)	0.70 (0.22)	0.54 (0.26)	0.24 (0.31)	-0.14 (0.24)
USA	0.92 (0.12)	0.85 (0.14)	0.77 (0.15)	0.65 (0.17)	0.44 (0.22)	0.14 (0.16)
Zimbabwe	0.92 (0.12)	0.85 (0.13)	0.75 (0.13)	0.64 (0.14)	0.48 (0.19)	0.22 (0.18)

Supplement IV) Mean HU estimates generated using country-specific value sets applied to appropriate populations, and stratified by mRS at 3 months (HU displayed as mean (StdDev))

Populations Analysed	Value Set Applied	Modified Rankin Scale Score at day 90 at day 90					
		0	1	2	3	4	5
**Australia & New Zealand (n=80)**	**Australia**	0.96 (0.05)	0.82 (0.18)	0.77 (0.18)	0.58 (0.20)	0.49 (0.17)	0.02 (0.14)
**China, Hong Kong, Taiwan (n=138)**	**China**	0.95 (0.08)	0.87 (0.14)	0.79 (0.15)	0.60 (0.16)	0.34 (0.19)	0.17 (0.21)
**Finland, Norway, Sweden (n=40)**	**Denmark**	0.90 (0.12)	0.82 (0.09)	0.92 (0.12)	0.60 (0.16)	0.37 (0.22)	-0.22 (0.28)
**Germany, Italy, Austria, Greece, Switzerland (n=325)**	**Germany**	0.98 (0.04)	0.93 (0.13)	0.86 (0.17)	0.65 (0.24)	0.46 (0.24)	0.03 (0.15)
**Netherlands, France, Belgium (n=215)**	**Netherlands**	0.89 (0.10)	0.83 (0.19)	0.75 (0.15)	0.61 (0.21)	0.34 (0.30)	0.12 (0.21)
**Poland, Hungary, Bulgaria,**	**Poland**	0.96 (0.06)	0.90 (0.10)	0.83 (0.10)	0.73 (0.14)	0.39 (0.29)	0.01 (0.26)

**Czech Republic, Slovak Republic, Russia (n=1133)**							
**Singapore, Malaysia, Philippines (n=160)**	**Singapore**	0.96 (0.09)	0.81 (0.21)	0.47 (0.26)	0.24 (0.33)	-0.23 (0.29)	-0.48 (0.17)
**South Korea (n=32)**	**South Korea**	0.93 (0.04)	0.86 (0.09)	0.80 (0.07)	0.66 (0.17)	0.41 (0.25)	0.27 (0.46)
**Spain, Portugal (n=377)**	**Spain**	0.88 (0.17)	0.86 (0.16)	0.67 (0.27)	0.48 (0.30)	0.08 (0.40)	-0.33 (0.28)
**UK (n=70)**	**UK**	0.81 (0.13)	0.90 (0.11)	0.72 (0.15)	0.42 (0.27)	0.19 (0.29)	-0.20 (0.19)
**USA, Canada, Brazil, Mexico, Argentina, Chile, Israel (n=1246)**	**USA**	0.89 (0.16)	0.83 (0.15)	0.75 (0.16)	0.65 (0.19)	0.44 (0.22)	0.15 (0.14)
**South Africa (n=43)**	**Zimbabwe**	0.94 (0.12)	0.84 (0.14)	0.77 (0.13)	0.58 (0.10)	0.53 (0.20)	0.25 (0.05)
